# Synthesis of *N*-substituted Acridinediones and Polyhydroquinoline Derivatives in Refluxing Water

**DOI:** 10.3390/molecules17055339

**Published:** 2012-05-07

**Authors:** Jing-Jing Xia, Ke-Hua Zhang

**Affiliations:** School of Materials and Chemical Engineering, Anhui University of Architecture, Hefei 230601, China; E-Mail: zhangkehua@aiai.edu.cn

**Keywords:** acridinedione, polyhydroquinoline, one-pot reaction, water

## Abstract

Acridinediones were synthesized by the one-pot Hantzsch condensation of an aromatic aldehyde, 5,5-dimethyl-1,3-cyclohexanedione, and aniline/4-methylaniline in refluxing water. This method has then been extended to the four-component reaction of an aromatic aldehyde, 5,5-dimethyl-1,3-cyclohexanedione, ethyl acetoacetate and ammonium acetate for the synthesis of polyhydroquinoline derivatives. This is an environmentally friendly and efficient procedure providing good to excellent yields.

## 1. Introduction

1,4-Dihydropyridines as analogues of nicotinamide adenine dinucleotide (NADH) coenzymes exhibit a wide range of biological activities, such as calcium channel blocking, and today they are widely used in pharmacology [1]. Acridines which possess the 1,4-dihydropyridine parent nucleus have interesting pharmaceutical properties such as a positive iontropic effects promoting the entry of calcium to the intracellular space [2], and 1,8-(2*H*,5*H*)-acridinediones are known as laser dyes [3].

1,8-(2*H*,5*H*)-Acridinediones were synthesized by the adoption of the Hantzsch procedure, *i.e.*, by the thermal reaction of 5,5-dimethyl-1,3-cyclohexanedione (dimedone) with an aldehyde and ammonia. Most of the methods reported previously usually require long reaction times, afford 1,4-dihydropyridines in relatively low yield, and suffer from utilizing harmful organic solvents in most cases [4,5,6,7,8,9,10,11].

On the other hand, with the increasing environmental concerns, green chemistry has attracted intensive attention in recent years. Multicomponent one-pot reactions as a kind of economical and efficient procedure has been widely used for the synthesis of heteroatom-containing compounds [12,13,14]. Moreover, organic reactions in water as the reaction medium, which represents a clean, economical, and environmental-safe protocol, has attracted considerable attention. We have already reported the one-pot synthesis of 10-unsubstituted 1,8-(2*H*,5*H*)-acridinediones by the thermal reaction of 5,5-dimethyl-1,3-cyclohexanedione (dimedone) with an aldehyde and ammonium acetate in pure water without any additives [15]. In this paper, ammonium acetate has been replaced by aniline and 4-methylaniline for the synthesis of *N*-substituted 1,8-(2*H*,5*H*)-acridinediones by the Hantzsch reaction in refluxing water.

## 2. Results and Discussion

1,8-(2*H*,5*H*)-Acridinediones were synthesized by the one-pot three-component Hantzsch condensation for a designated time of paraformaldehyde, dimedone and NH_4_HCO_3_, or NH_4_OAc, or aniline, or 4-methyl-aniline in refluxing water without any additives. The reaction times and yields are listed in Table 1.

From Table 1, one can see that the reaction yields of ammonium compounds (entry 1 and 2) are higher than for aromatic amines (entry 3 and 4). This is probably due to the lower solubility of the aromatic amines in water and lower reactivity than that of the ammonium compounds. It should be mentioned that the amount of the amine in the reactions was different. When an aromatic amine was used (entry 3 and 4), the molar ratio of **1**, **2**, and **3** was 1:2:1; when an ammonium compound was used (entry 1 and 2), the molar ratio of **1**, **2**, and **3** was 1:2:4. The increasing of the dosage of ammonium compound is because of the easy resolvability.

In order to obtain a higher yield, we tried to add a phase-transfer catalyst (PTC) to the reaction mixtures. Cetyltrimethylammonium bromide (CTAB) was previously applied by us to the aqueous Michael reaction of dimedone with chalcones [16], and now in this Hantzsch condensation of aldehyde, dimedone and aniline, addition of 10% mol of CTAB produced *N*-substituted 1,8-(2*H*,5*H*)-acridinediones in much better yield. The reaction yields, as well as the melting points for the Hantzsch reactions with the molar ratio of aromatic aldehydes **1**, dimedone (**2**), and aniline or 4-methylaniline **3**, and CTAB as 1:2:1:0.1 in refluxing water are listed in Table 2. Reaction times are all 90 min. In our protocol, no organic solvents were used during the reaction process. Furthermore, since the product is solid and precipitates out from the reaction mixtures, the work-up procedure involves simple filtration. The desired products of high purity were obtained by column chromatography or recrystallization.

From Table 2, one can see that all these reactions gave good to excellent yields, reflecting that dimedone has high solubility in water and high reactivity. The yields of *N*-substituted acridinediones for the aromatic aldehydes with an electron-withdrawing group are higher than those for aromatic aldehydes with electron-donating groups. We also tried 3,4-dimethoxybenzaldehyde, but the reaction yield is lower than 50%.

We have attempted to extend the above methods to different 1,3-dicarbonyl compounds for the synthesis of unsymmetrically substituted 1,4-dihydropyridines such as polyhydroquinoline derivatives. Ethyl acetoacetate and 1,3-cyclohexanedione smoothly undergo a four-component reaction with aromatic aldehyde and ammonium acetate to produce polyhydroquinoline derivatives in refluxing water catalyzed by CTAB. Because of the lower activity for the synthesis of unsymmetrically substituted 1,4-dihydropyridines than the symmetrically substituted 1,4-dihydropyridines, ammonium acetate was used to replace the aromatic amines. The reaction yields, as well as the melting points for the Hantzsch reaction with the molar ratio of aromatic aldehydes **1**, dimedone (**2**), ethyl acetoacetate (**6**), ammonium acetate (**3**), and CTAB as 1:1:1:4:0.1 in refluxing water are listed in Table 3. Reaction times are all 90 min.

Among the acridinediones and polyhydroquinoline products **5c** and **5f** were unknown compounds and were characterized by their melting point, IR, ^1^H-NMR, ^13^C-NMR spectra and elemental analysis. Structures of known compounds were confirmed by comparison of their melting points and ^1^H-NMR spectra with the reported data. It should be mentioned that the melting point of **5d** was obviously higher than the reported in the literature [17]. However, our measured IR, ^1^H-NMR, ^13^C-NMR spectral data and elemental analysis are consistent with the series of acridinediones products, exhibiting the same identities. The different mp might be ascribed to the presence of an impurity or solvent in product **5d** from the reported literature, because even a trace amount could have a great effect on the m.p. of a compound with high m.p.

## 3. Experimental 

### 3.1. General

^1^H-NMR spectra were recorded on a Bruker Avance-400 (400 MHz) spectrometer (Bruker, Switzerland), and chemical shifts (δ) are reported in parts per million relative to tetramethylsilane and coupling constants (*J*) in Hz. Splitting patterns are designated as s, singlet; d, doublet; br, broad. ^13^C-NMR spectra were recorded on the same spectrometer (at 100 MHz) with complete proton decoupling, and chemical shifts are reported in parts per million relative to the solvent resonance used as the internal standard (CDCl_3_, δ 77.16 ppm; DMSO-d6, δ 39.52 ppm). IR spectra were taken on a Bruker Vector-22 spectrometer (Bruker, Switzerland) in KBr pellets and are reported in cm^−1^. Melting points were determined on an XT-4 apparatus (Beijing Tech Instrument Co., Beijing, China). Analytical TLC and column chromatography were performed on silica gel GF254 and silica gel H60, respectively.

### 3.2. Typical Procedure for the Synthesis of Acridinediones: 3,3,6,6-Tetramethyl-9,10-diphenyl-3,4,6,7,9,10-hexahydro-1,8-(2H,5H)-acridinedione *(**5a**)*

A mixture of **1a** (106.1 mg, 1 mmol), **2** (280.4 mg, 2 mmol), **3c** (93.1 mg, 1 mmol) and CTAB (36.4 mg, 0.1 mmol) in water (4 mL) was vigorously stirred under reflux. The reaction was completed after 90 min, as monitored by TLC. Due to the high yields of **5a**, the work-up procedure involved simple filtration and washing twice with water (10 mL). The obtained solid products were nearly pure. The desired product of high purity were further achieved by column chromatography with petroleum ether/ethyl acetate or recrystallization from 75% aqueous ethanol.

*3*,*3*,*6*,*6-Tetramethyl-9-(4-cyanophenyl)-10-phenyl-3*,*4*,*6*,*7*,*9*,*10-hexahydro-1*,*8-(2H*,*5H)-acridinedione* (**5c**). IR (KBr): υ 2956 (s), 2872 (m), 2223 (m), 1643 (s), 1576 (s), 1492 (m), 1362 (s), 1297 (w), 1262 (m), 1174 (w), 1144 (m), 1122 (w), 1000 (m), 851 (s), 704 (s), 569 (s) cm^−1^; ^1^H-NMR (CDCl_3_): δ 0.79 (s, 6H, CH_3_), 0.95 (s, 6H, CH_3_), 1.85 (d, *J* = 17.5 Hz, 2H, CH_2_), 2.11 (d, *J* = 17.5 Hz, 2H, CH_2_), 2.12 (d, *J* = 16.2 Hz, 2H, CH_2_), 2.21 (d, *J* = 16.2 Hz, 2H, CH_2_), 5.31 (s, 1H, CH), 7.25 (d, *J* = 8.0 Hz, 2H, ArH), 7.54 (d, *J* = 8.6 Hz, 2H, ArH), 7.58 (d, *J* = 8.0 Hz, 2H, ArH), 7.59 (m, 3H, ArH); ^13^C-NMR (CDCl_3_): δ 195.8, 151.6, 150.6, 138.6, 131.9, 129.7, 128.8, 119.3, 113.4, 109.5, 50.1, 41.8, 33.7, 32.4, 29.6, 26.7; Anal. Calcd. for C_30_H_30_N_2_O_2_: C, 79.97; H, 6.71; N, 6.22; Found: C, 79.97; H, 6.80; N, 6.17.

*3*,*3*,*6*,*6-Tetramethyl-9-(4-nitrophenyl)-10-phenyl-3*,*4*,*6*,*7*,*9*,*10-hexahydro-1*,*8-(2H*,*5H)-acridinedione* (**5d**). IR (KBr): υ 2956 (m), 1635 (s), 1594 (w), 1514 (m), 1349 (s), 1224 (m), 1176 (w), 1144 (w), 1113 (w), 1003 (m), 864 (w), 830 (w), 703 (m), 572 (w), 513 (w) cm^−1^; ^1^H-NMR (CDCl_3_): δ 0.72 (s, 6H, CH_3_), 0.88 (s, 6H, CH_3_), 1.77 (d, *J* = 17.5 Hz, 2H, CH_2_), 2.03 (d, *J* = 17.5 Hz, 2H, CH_2_), 2.04 (d, *J* = 16.3 Hz, 2H, CH_2_), 2.14 (d, *J* = 16.3 Hz, 2H, CH_2_), 5.28 (s, 1H, CH), 7.18 (d, *J* = 8.2 Hz, 2H, ArH), 7.53 (d, *J* = 8.2 Hz, 2H, ArH), 7.54 (s, 1H, ArH), 7.55 (d, *J* = 8.8 Hz, 2H, ArH), 8.05 (d, *J* = 8.8 Hz, 2H, ArH); ^13^C-NMR (CDCl_3_): δ 195.6, 153.6, 150.4, 146.2, 138.7, 129.7, 128.8, 123.5, 113.6, 50.1, 41.9, 33.6, 32.4, 29.6, 26.7; Anal. Calcd. for C_29_H_30_N_2_O_4_: C, 74.02; H, 6.43; N, 5.95; Found: C, 73.63; H, 6.43; N, 5.91.

*3*,*3*,*6*,*6-Tetramethyl-9-(3*,*4-dichlorophenyl)-10-phenyl-3*,*4*,*6*,*7*,*9*,*10-hexahydro-1*,*8-(2H,5H)-acridine-dione* (**5f**). IR (KBr): υ 2962 (m), 2948 (m), 1650 (s), 1638 (s), 1573 (m), 1471 (m), 1360 (s), 1224 (s), 1143 (m), 1027 (w), 1002 (w), 878 (m), 704 (s), 574 (m) cm^−1^; ^1^H-NMR (CDCl_3_): δ 0.80 (s, 6H, CH_3_), 0.93 (s, 6H, CH_3_), 1.83 (d, *J* = 17.6 Hz, 2H, CH_2_), 2.08 (d, *J* = 17.6 Hz, 2H, CH_2_), 2.17 (m, 4H, CH_2_), 5.21 (s, 1H, CH), 7.22 (m, 2H, ArH), 7.28 (m, 2H, ArH), 7.50 (s 1H, ArH), 7.56 (m, 3H, ArH); ^13^C-NMR (CDCl_3_): δ 195.7, 150.3, 146.6, 138.7, 131.9, 130.0, 129.9, 129.6, 127.6, 113.8, 50.1, 41.8, 32.4, 29.7, 26.8; Anal. Calcd. for C_29_H_29_Cl_2_NO_2_: C, 70.44; H, 5.91; N, 2.83; Found: C, 70.40; H, 6.01; N, 2.78.

## 4. Conclusions

In summary, acridinediones and polyhydroquinoline derivatives were synthesized by the one-pot Hantzsch condensation of an aromatic aldehyde, 5,5-dimethyl-1,3-cyclohexanedione, ethyl acetoacetate and amine. It represents a straightforward protocol for the eco-friendly and efficient synthesis of a series of 1,4-dihydropyridines with potential biological activities.

## Figures and Tables

**Table 1:**
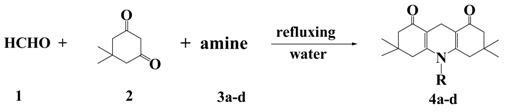
Reaction time and yield for the reaction of different amines/ammonium salts.

Entry	Amine	Reaction time/min	Product	R	Yield / % ^a^
1	NH_4_OAc	40 min	**4a**	H	86
2	NH_4_HCO_3_	40 min	**4b**	H	83
3	C_6_H_5_-NH_2_	90 min	**4c**	C_6_H_5_	71
4	4-CH_3_-C_6_H_5_-NH_2_	90 min	**4d**	4-CH_3_-C_6_H_4_	69

^a^ Isolated yield of the pure product recrystallized from 75% aqueous ethanol.

**Table 2:**
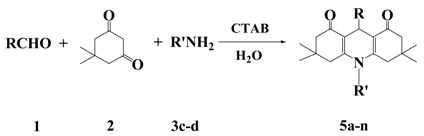
Yields and melting points for the one-pot synthesis of *N*-substituted acridinediones.

Entry	R	R’	Product	Yield / % ^a^	m.p. (lit.) / °C
1	C_6_H_5_	C_6_H_5_	**5a**	80	220–222 (200–205) [17]
2	4-Cl-C_6_H_4_	C_6_H_5_	**5b**	86	243–245 (233–235) [18]
3	4-CN-C_6_H_4_	C_6_H_5_	**5c**	88	265–267
4	4-NO_2_-C_6_H_4_	C_6_H_5_	**5d**	85	281–282 (216–218) [17]
5	3- NO_2_-C_6_H_4_	C_6_H_5_	**5e**	84	272–274 (276–278) [19]
6	3,4- Cl_2_-C_6_H_3_	C_6_H_5_	**5f**	90	274–275
7	4-CH_3_O-C_6_H_4_	C_6_H_5_	**5g**	70	291–293 (290–291) [20]
8	C_6_H_5_	4-CH_3_-C_6_H_4_	**5h**	81	261–263 (264–266) [21]
9	4-Cl-C_6_H_4_	4-CH_3_-C_6_H_4_	**5i**	85	270–272 (271–272) [21]
10	4-CN-C_6_H_4_	4-CH_3_-C_6_H_4_	**5j**	88	268–270 (273–275) [21]
11	4-NO_2_-C_6_H_4_	4-CH_3_-C_6_H_4_	**5k**	83	>300 (>300) [22]
12	3-NO_2_-C_6_H_4_	4-CH_3_-C_6_H_4_	**5l**	85	281–283 (283–284) [23]
13	3,4-Cl_2_-C_6_H_3_	4-CH_3_-C_6_H_4_	**5m**	88	253–255 (250–252) [23]
14	4-CH_3_O-C_6_H_4_	4-CH_3_-C_6_H_4_	**5n**	72	280–282 (281–283) [23]

^a^ Isolated yield of the pure product recrystallized from 75% aqueous ethanol.

**Table 3:**
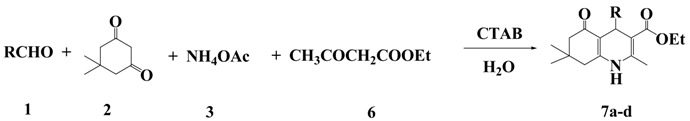
Yields and melting points for the one-pot synthesis of polyhydroquinoline derivatives.

Entry	R	Product	Yield / % ^a^	m.p. (lit.) / °C
1	C_6_H_5_	**7a**	85	224–226 (228–229) [24]
2	4-CH_3_O-C_6_H_4_	**7b**	81	257–259 (260–262) [24]
3	4-Cl-C_6_H_4_	**7c**	90	244–266 (245–246) [24]
4	4-NO_2_-C_6_H_4_	**7d**	88	242–244 (241–242) [24]

^a^ Isolated yield of the pure product recrystallized from 75% aqueous ethanol.

## Supplementary Materials

Supplementary materials can be accessed at: http://www.mdpi.com/1420-3049/17/5/5339/s1.
