# Supplementary material for: Synthesis of N-substituted Acridinediones and Polyhydroquinoline Derivatives in Refluxing Water
Source: Molecules. 2012 May 7;17(5):5339–45. doi: 10.3390/molecules17055339 (PMC6268734; doi:10.3390/molecules17055339)
Supplement: Supplementary file 1 [file molecules-17-05339-s001.pdf]

Molecules **2012**, *17*, 5339-5345; doi:10.3390/molecules17055339

OPEN ACCESS
molecules
ISSN 1420-3049
www.mdpi.com/journal/molecules

Communication

Synthesis of *N*-substituted Acridinediones and Polyhydroquinoline Derivatives in Refluxing Water

Jing-Jing Xia * and Ke-Hua Zhang

School of Materials and Chemical Engineering, Anhui University of Architecture, Hefei, 230601, China; E-Mail: zhangkehua@aiai.edu.cn

* Authors to whom correspondence should be addressed; E-Mail: xiajj@aiai.edu.cn; Tel.: +86-551-382-8100.

Received: 13 March 2012; in revised form: 24 April 2012 / Accepted: 28 April 2012 / Published: 7 May 2012

Supporting Information

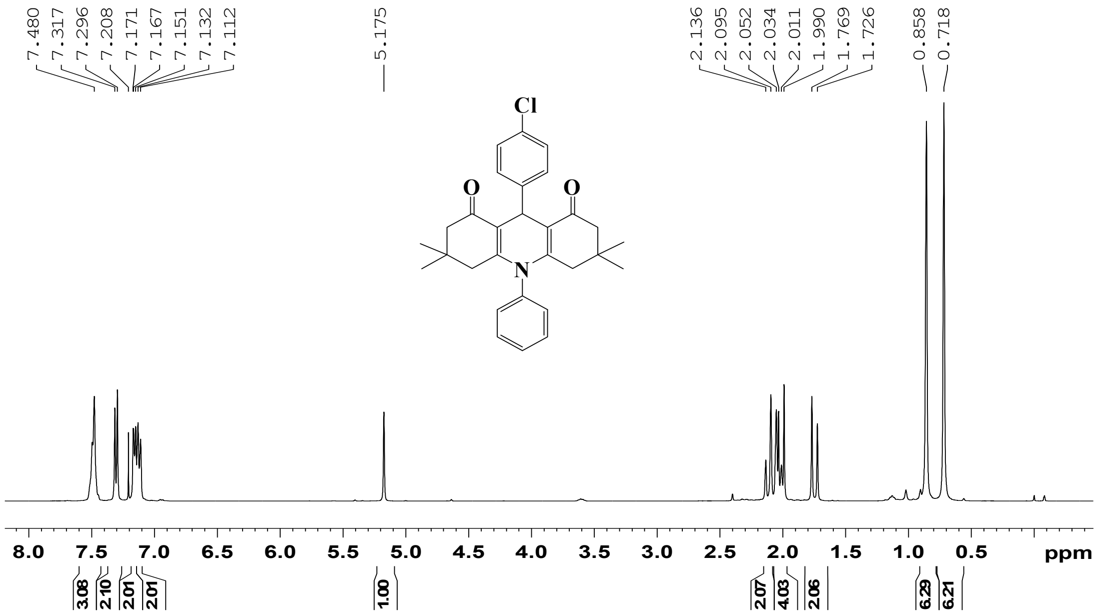

(**5b**): ^{1}H-NMR ($CDCl_3$): δ 0.72 (s, 6H, CH_3), 0.86 (s, 6H, CH_3), 1.75 (d, J = 17.5 Hz, 2H, CH_2), 2.01 (d, J = 17.5 Hz, 2H, CH_2), 2.03 (d, J = 16.2 Hz, 2H, CH_2), 2.12 (d, J = 16.2 Hz, 2H, CH_2), 5.18 (s, 1H, CH), 7.12 (d, J = 8.3 Hz, 2H, ArH), 7.16 (d, J = 7.8 Hz, 2H, ArH), 7.30 (d, J = 8.3 Hz, 2H, ArH), 7.48 (m, 3H, ArH).

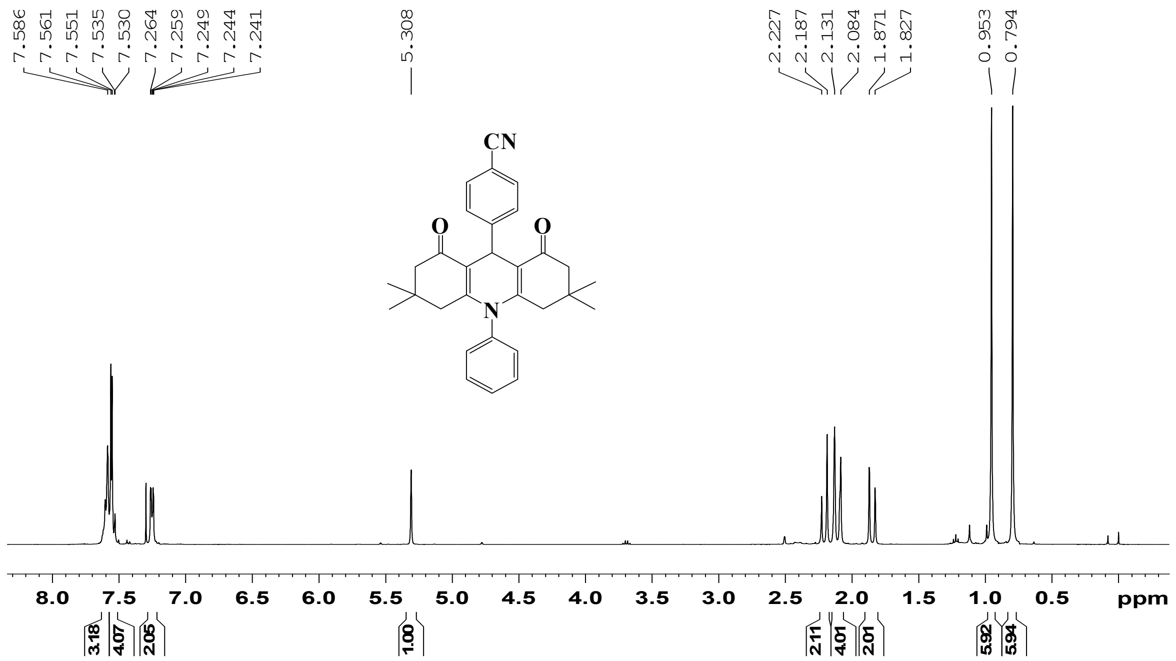

(**5c**): ^{1}H-NMR ($CDCl_3$): δ 0.79 (s, 6H, CH_3), 0.95 (s, 6H, CH_3), 1.85 (d, J = 17.5 Hz, 2H, CH_2), 2.11 (d, J = 17.5 Hz, 2H, CH_2), 2.12 (d, J = 16.2 Hz, 2H, CH_2), 2.21 (d, J = 16.2 Hz, 2H, CH_2), 5.31 (s, 1H, CH), 7.25 (d, J = 8.0 Hz, 2H, ArH), 7.54 (d, J = 8.6 Hz, 2H, ArH), 7.58 (d, J = 8.0 Hz, 2H, ArH), 7.59 (m, 3H, ArH).

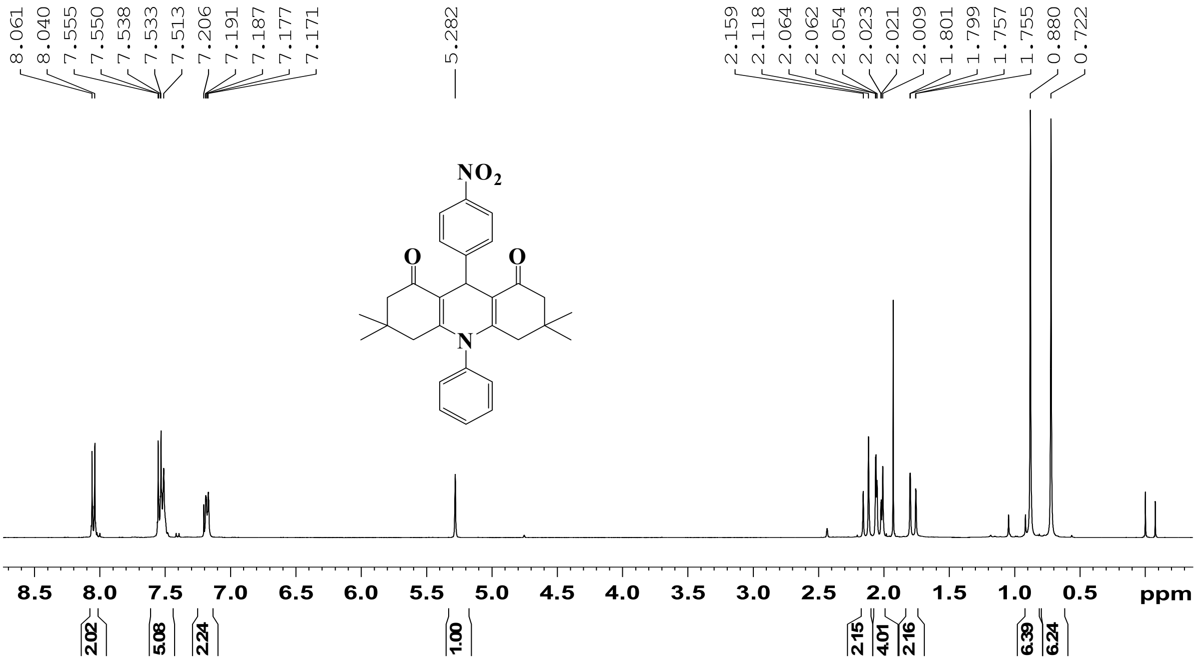

(**5d**): ^{1}H-NMR ($CDCl_3$): δ 0.72 (s, 6H, CH_3), 0.88 (s, 6H, CH_3), 1.77 (d, J = 17.5 Hz, 2H, CH_2), 2.03 (d, J = 17.5 Hz, 2H, CH_2), 2.04 (d, J = 16.3 Hz, 2H, CH_2), 2.14 (d, J = 16.3 Hz, 2H, CH_2), 5.28 (s, 1H, CH), 7.18 (d, J = 8.2 Hz, 2H, ArH), 7.53 (d, J = 8.2 Hz, 2H, ArH), 7.54 (s, 1H, ArH), 7.55 (d, J = 8.8 Hz, 2H, ArH), 8.05 (d, J = 8.8 Hz, 2H, ArH).

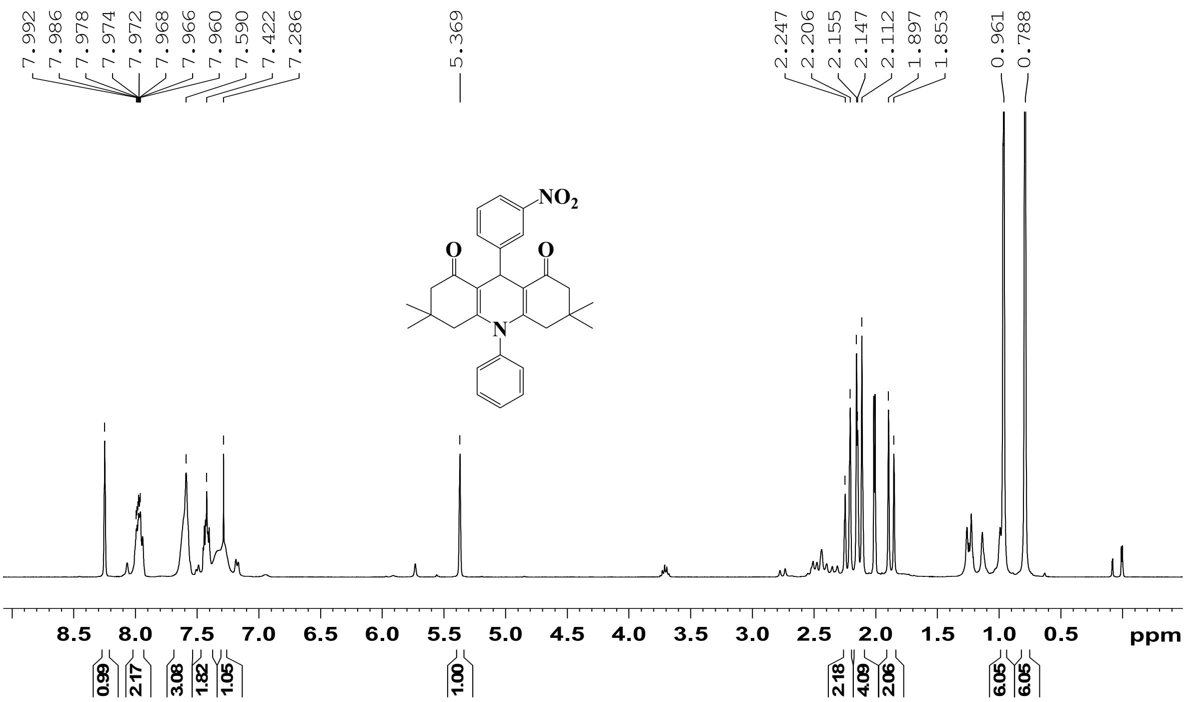

(**5e**): ^{1}H-NMR ($CDCl_3$): δ 0.79 (s, 6H, CH_3), 0.96 (s, 6H, CH_3), 1.87 (d, J = 17.6 Hz, 2H, CH_2), 2.12 (d, J = 16.2 Hz, 2H, CH_2), 2.13 (d, J = 17.6 Hz, 2H, CH_2), 2.22 (d, J = 16.2 Hz, 2H, CH_2), 5.37 (s, 1H, CH), 7.29 (m, 1H, ArH), 7.42 (m, 2H, ArH), 7.59 (m, 3H, ArH), 7.97 (m, 2H, ArH), 8.24 (s, 1H, ArH).

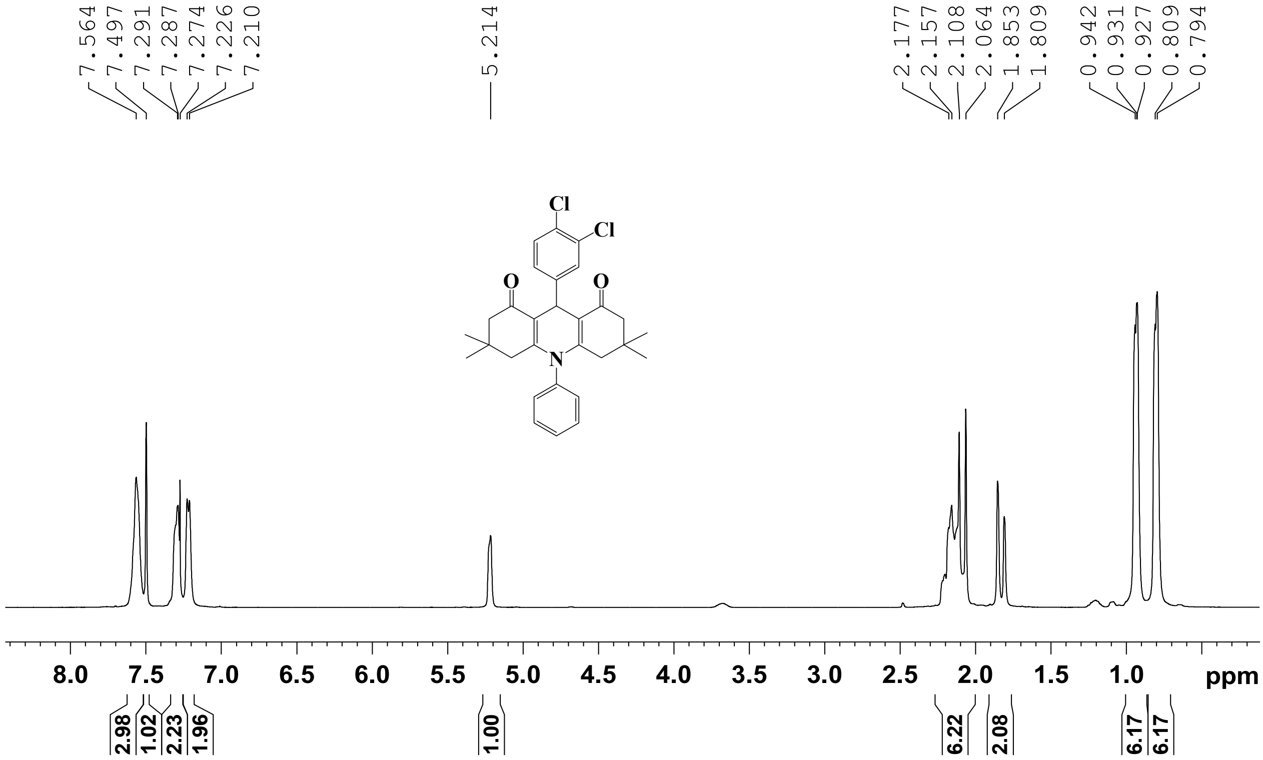

(**5f**): ^{1}H-NMR ($CDCl_3$): δ 0.80 (s, 6H, CH_3), 0.93 (s, 6H, CH_3), 1.83 (d, J = 17.6 Hz, 2H, CH_2), 2.08 (d, J = 17.6 Hz, 2H, CH_2), 2.17 (m, 4H, CH_2), 5.21 (s, 1H, CH), 7.22 (m, 2H, ArH), 7.28 (m, 2H, ArH), 7.50 (s 1H, ArH), 7.56 (m, 3H, ArH).

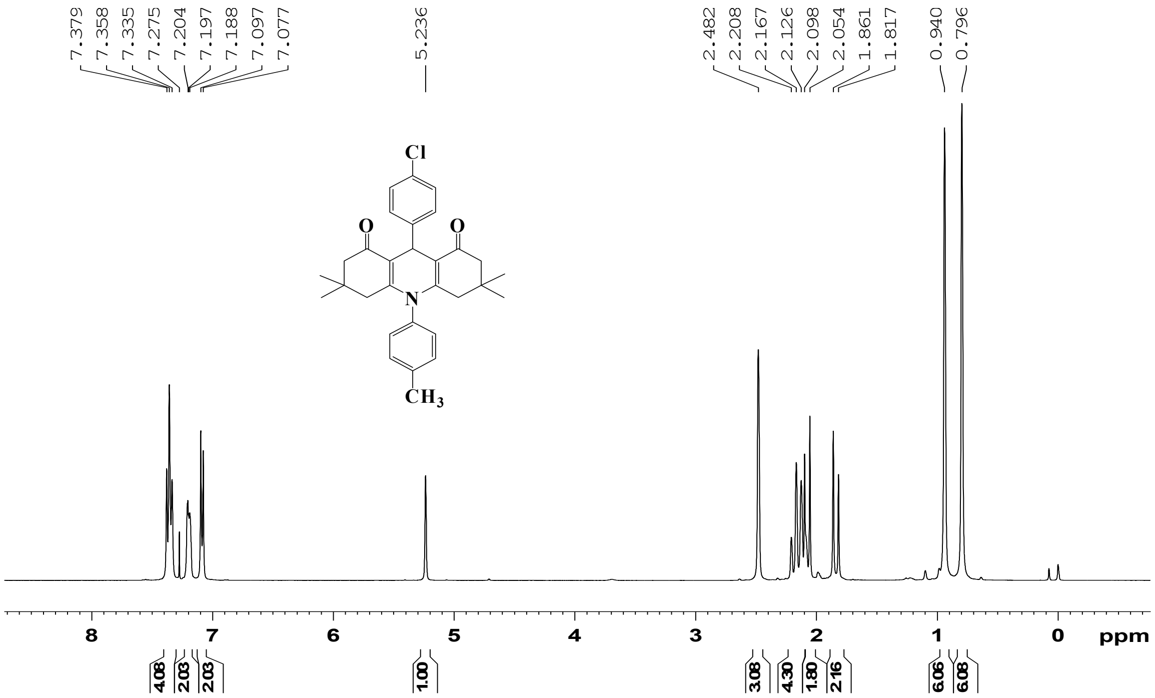

(**5i**): ^{1}H-NMR ($CDCl_3$): δ 0.80 (s, 6H, CH_3), 0.94 (s, 6H, CH_3), 1.84 (d, J = 17.5 Hz, 2H, CH_2), 2.07 (d, J = 17.5 Hz, 2H, CH_2), 2.10 (d, J = 16.3 Hz, 2H, CH_2), 2.19 (d, J = 16.3 Hz, 2H, CH_2), 2.48 (s, 3H, CH_3), 5.24 (s, 1H, CH), 7.08 (d, J = 8.3 Hz, 2H, ArH), 7.19 (d, J = 9.2 Hz, 2H, ArH), 7.34 (d, J = 9.2 Hz, 2H, ArH), 7.37 (d, J = 8.3 Hz, 2H, ArH).

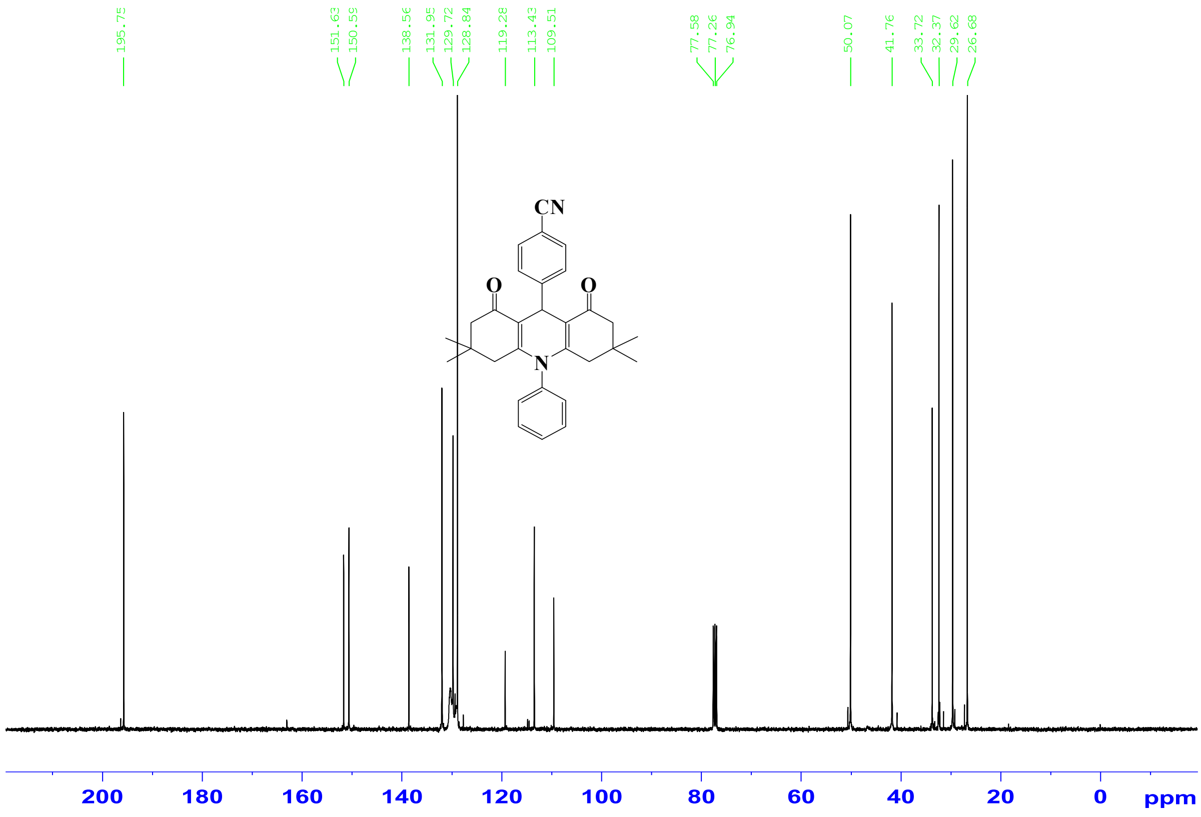

(**5c**): ^{13}C-NMR ($CDCl_3$): δ 195.8, 151.6, 150.6 138.6, 131.9, 129.7, 128.8, 119.3, 113.4, 109.5, 50.1, 41.8, 33.7, 32.4, 29.6, 26.7.

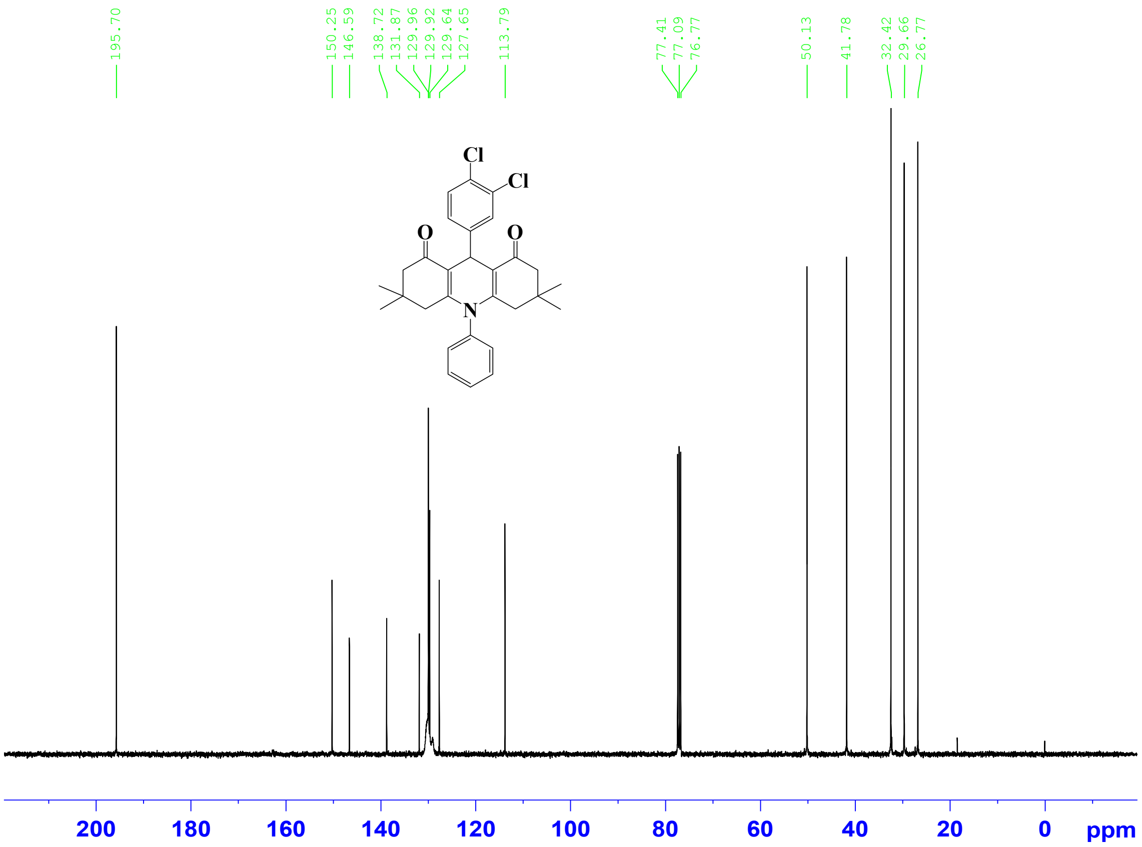

(**5f**): ^{13}C-NMR ($CDCl_3$): δ 195.7, 150.3, 146.6, 138.7, 131.9, 130.0, 129.9, 129.6, 127.6, 113.8, 50.1, 41.8, 32.4, 29.7, 26.8.

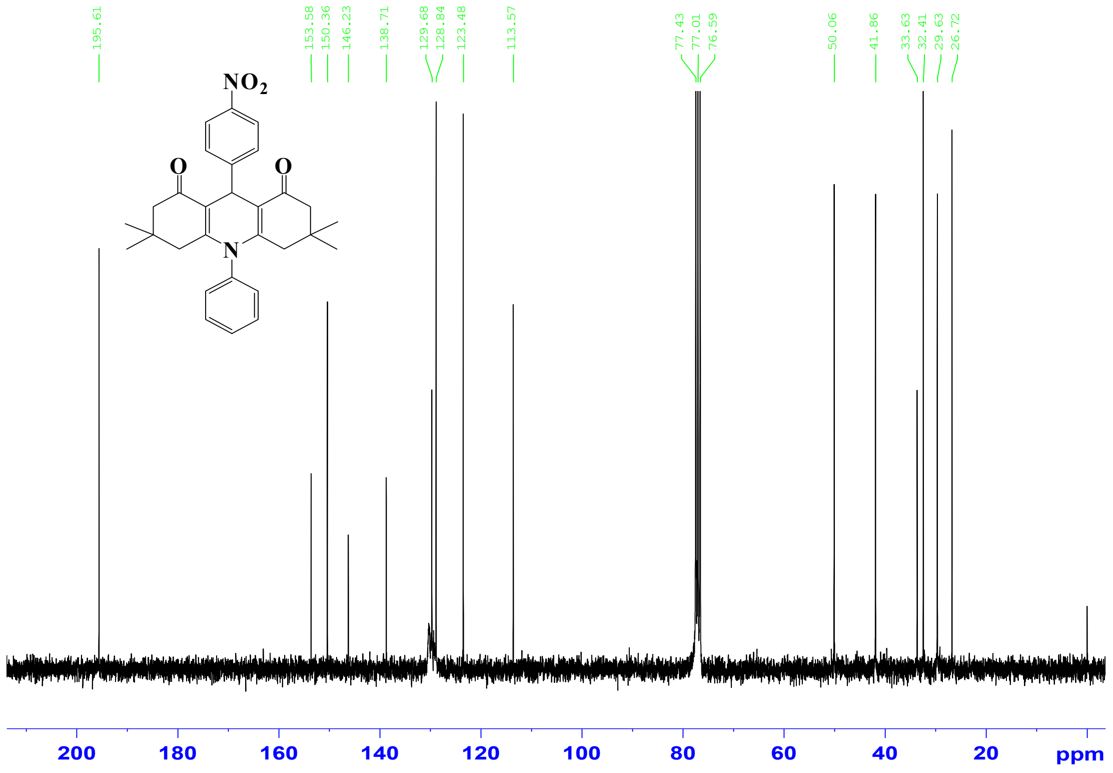

(**5d**): ^{13}C-NMR ($CDCl_3$): δ 195.6, 153.6, 150.4, 146.2, 138.7, 129.7, 128.8, 123.5, 113.6, 50.1, 41.9, 33.6, 32.4, 29.6, 26.7.

Elemental Analysis

Document Untitled (varioELcube) from: --.--.-- (modified)

中国科学技术大学 理化科学实验中心
元素分析仪: Elementar vario EL cube
炉温：燃烧管 950度，还原管 550度

Text report

	Name	Weight [mg]	N [%]	C [%]	H [%]	N Factor	C Fac...	H Factor	Info	Date Time
17	xia-4	2.845	2.78	70.40	6.01	1.0007	1.0221	0.9910		09.09.2011 11:12
18	xia-5	2.219	6.17	79.97	6.80	1.0007	1.0221	0.9910		09.09.2011 11:19

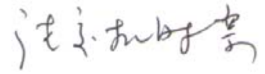

Name: eassuperuser, Access: varioELcube superuser 2011-9-9 14:11:26

varioEL cube V2.0.3 (9395a42)2011-01-27, CHN Mode, Ser. No.: 19111010
Elementar Analysensysteme GmbH

Page 1 (of 1)

Document Untitled (varioELcube) from: --.--.-- (modified)

中国科学技术大学 理化科学实验中心
元素分析仪: Elementar vario EL cube
炉温：燃烧管 950度，还原管 550度

Text report

	Name	Weight [mg]	N [%]	C [%]	H [%]	N Factor	C Fac...	H Factor	Date	Time
14	01-05-052	2.058	5.91	73.63	6.43	0.9933	1.0146	0.9904	11.01.2012	11:08

Name: eassuperuser, Access: varioELcube superuser 2012-1-11 15:21:27

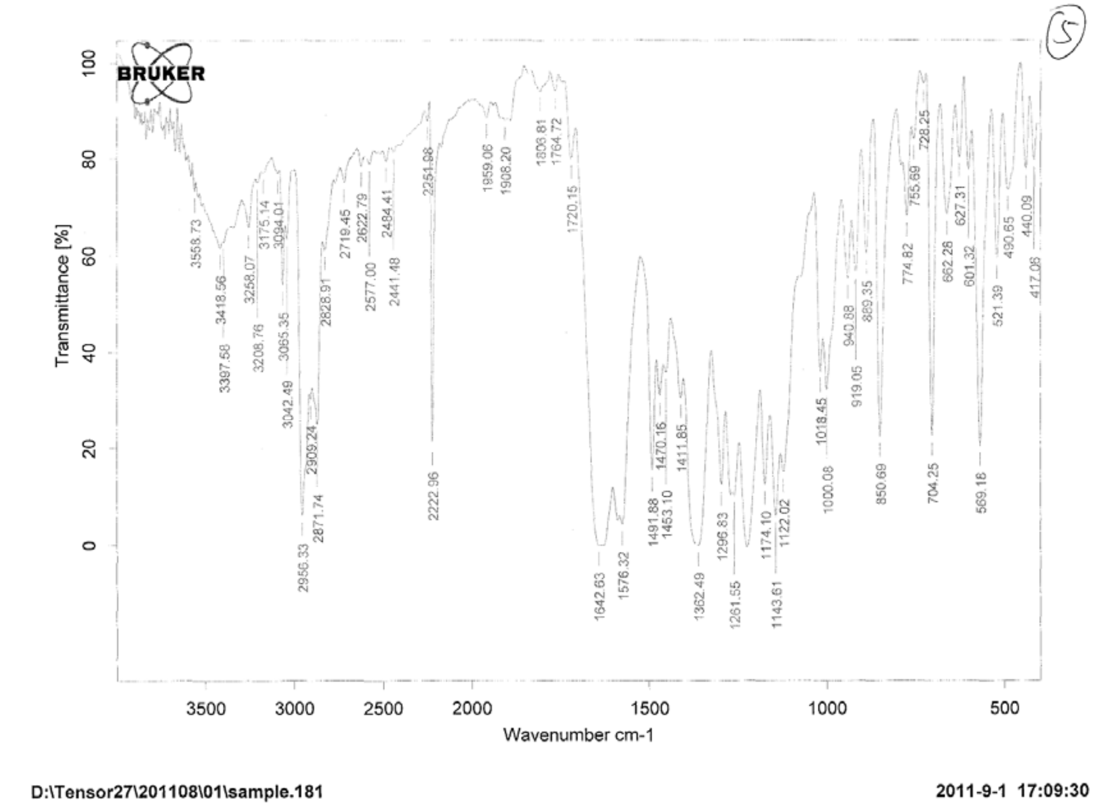

(**5c**): IR (KBr): υ 2956 (s), 2872 (m), 2223 (m), 1643 (s), 1576 (s), 1492 (m), 1362 (s), 1297 (w), 1262 (m), 1174 (w), 1144 (m), 1122 (w), 1000 (m), 851 (s), 704 (s), 569 (s) cm^{-1}.

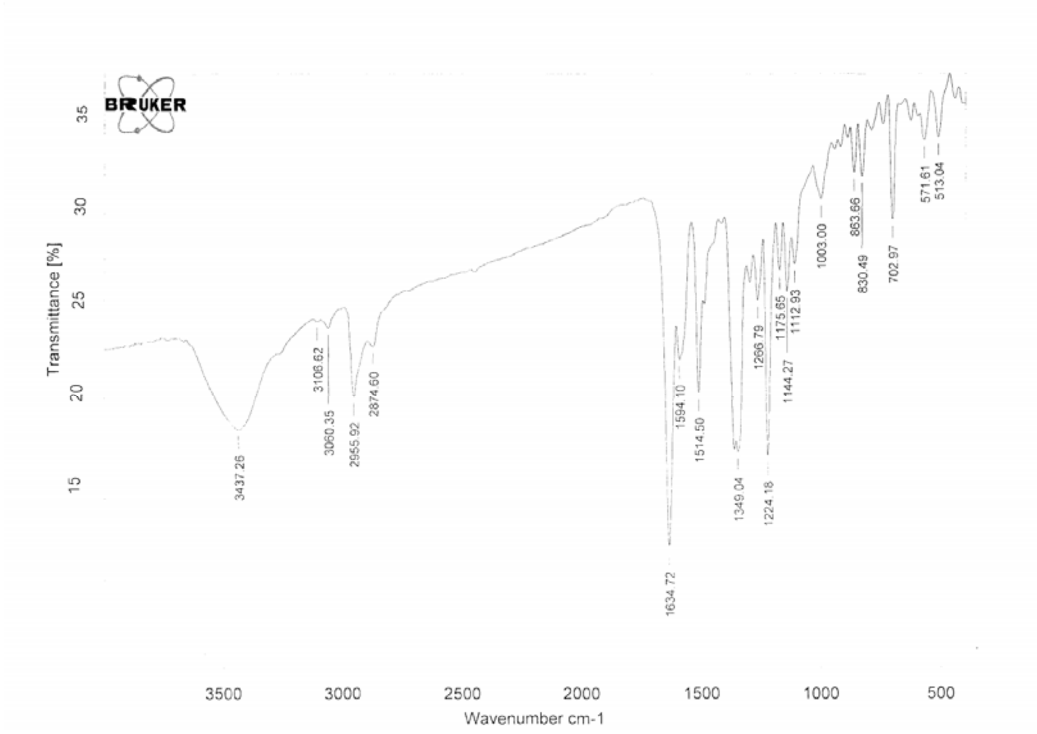

(**5d**): IR (KBr): υ 2956 (m), 1635 (s), 1594 (w), 1514 (m), 1349 (s), 1224 (m), 1176 (w), 1144 (w), 1113 (w), 1003 (m), 864 (w), 830 (w), 703 (m), 572 (w), 513 (w) cm^{-1}.

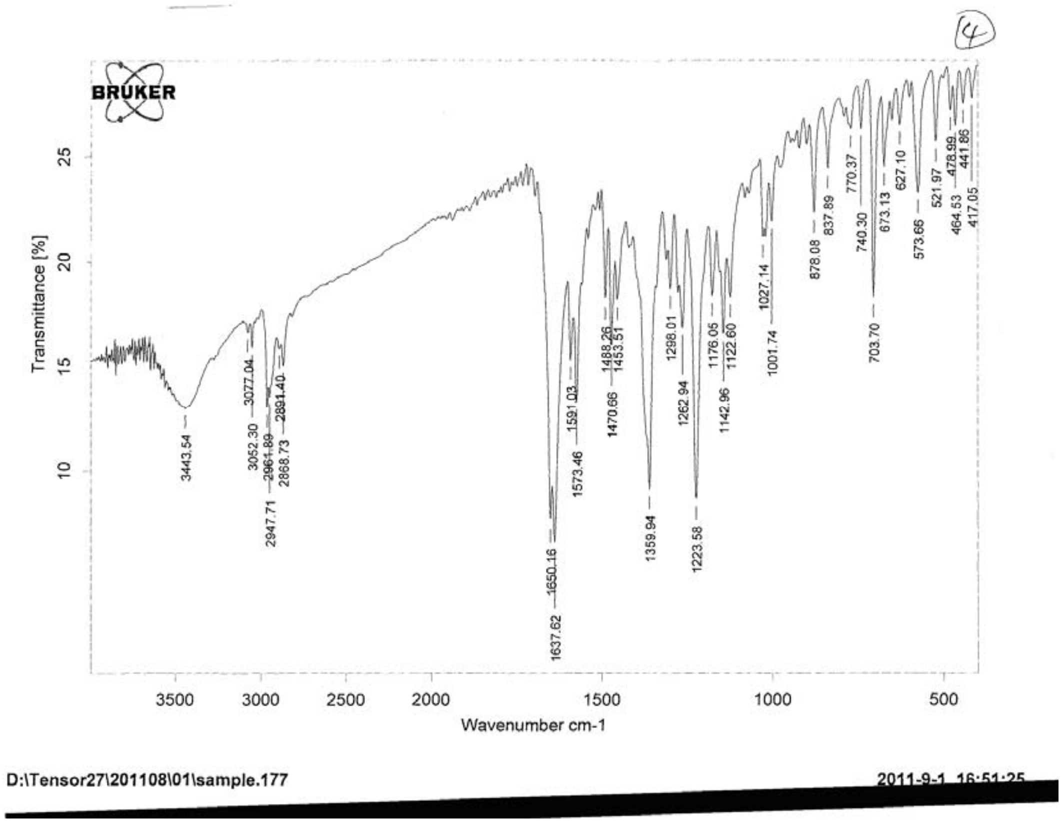

(**5f**): IR (KBr): υ 2962 (m), 2948 (m), 1650 (s), 1638 (s), 1573 (m), 1471 (m), 1360 (s), 1224 (s), 1143 (m), 1027 (w), 1002 (w), 878 (m), 704 (s), 574 (m) cm^{-1}.
